# Population dynamics of soil bacteria in some areas of Midnapore coastal belt, West Bengal, India

**DOI:** 10.1007/s13205-015-0361-y

**Published:** 2016-01-23

**Authors:** Syed Afrin Azmi, Soumendranath Chatterjee

**Affiliations:** Parasitology and Microbiology Research Laboratory, Department of Zoology, The University of Burdwan, Burdwan, West Bengal 713104 India

**Keywords:** Population dynamics, ANOVA, Shannon-Wiener Index, Simpson Index, PCA, AHC

## Abstract

In this present study the population dynamics of the soil bacteria of some coastal villages, namely Padima, Jatimati, Chanpabani, Palsandapur, Bhagibaharampur, Duttapur, Gangadharpur, Gobindabasan, Somaibasan of Digha, West Bengal, India, was determined. In these villages the aerobic heterotrophic, Gram-negative, spore-forming, starch-hydrolyzing, *Pseudomonas*, nitrate-reducing, denitrifying, asymbiotic N_2_ fixing, nitrifying, phosphate-solubilizing bacterial populations ranged from 1.22 to 2.67 × 10^6^, 0.09–1.63 × 10^5^, 1.53–3.68 × 10^5^, 2.22–4.06 × 10^5^, 0.02–0.04 × 10^5^, 0.35–1.33 × 10^5^, 0.07–0.82 × 10^5^, 0.58–2.50 × 10^5^, 0.13–2.35 × 10^5^, 0.05–1.9 × 10^5^ cfu/g dry soil, respectively. The organic carbon content of the soil samples ranged from 0.61 to 0.93 %. The available nitrogen and phosphate in the soils of the study area varied from 11.2 to 29.5 and 230.8–503.09 mg/kg, respectively. The one-way ANOVA revealed significant variations (*p* < 0.05) in the microbial diversity with respect to different locations of the study site. Shannon-Wiener and Simpson Index of the study areas ranged from 1.56 to 1.88 and 3.85–5.73, respectively. Jatimati showed comparatively higher diversity index among the villages of the study area. From principal component analysis, three components were extracted having the Eigen values of 3.541, 1.603 and 1.391, respectively. Agglometric hierarchial cluster analysis in respect of the number of different bacterial groups in different places of the study area showed that the denitrifying, nitrate reducing, asymbiotic nitrogen-fixing and spore-forming bacteria formed a cluster while *Pseudomonas* differed from them forming another cluster and nitrifying, Gram negative, phosphate-solubilising and starch-hydrolyzing bacteria formed another different cluster. This variation of the soil bacteria might be dependent on the microhabitat present in different locations of the study area.

## Introduction

In terrestrial and aquatic ecosystems, soil is an important abiotic component that regulates the formation of assemblage of several interacting organisms, including microbes. Microorganisms are ubiquitous in nature and influence all known ecosystems on earth (Atlas and Bertha 1998). The ubiquity of microorganisms is attributed mainly to their small size, easy dispersal, adaptation to diverse habitats and ability to utilize wide variety of substrates as nutrient source (Pandey et al. 2007). The soil-dwelling microbes can be referred as the “Biological engine” of the earth as they play a pivotal role in many fundamental nutrient cycling processes, soil structure dynamics, pollution degradation and regulation of different plant communities (Breure 2004). Microbes are also responsible for soil aeration and soil fertility which are among the crucial aspects of soil function. Production of soil organic matter increases the capacity of the soil to maintain its functional structure once it is formed. The soil organic matter is directly derived from the combined biological activity of plants, microbes, animals and abiotic factors. Soil microbial community mainly consists of five major groups, i.e. bacteria, actinomycetes, fungi, algae and protozoa (Holt 1986). Among them bacterial population is generally much higher than other groups (Alexander 1978). To understand the complexity of the interaction mediated by soil microbes, the evaluation of soil microbial diversity is essential. The determination of soil microbes is based on enumeration of laboratory culture of the isolates and also on 16S rDNA sequence analysis of the microbes (Seckbach 2000; Satyanarayana et al. 2005). Various works have been done on soil microbial diversity in different regions of India (Das and Dangar 2007, 2008; Chatterjee et al. 2007) but no such work on bacterial diversity has yet been done in the coastal areas of Digha, West Bengal, India. Coastal zone is the transitional zone between the terrestrial land and sea which are indeed unique places in our global geography, so various physical and topological parameters of the coastal areas should be maintainedd and observe periodically (Visalatchi and Raj Chandar 2012). Though the coastal areas of Digha is an important sea-shore region of India, it is neglected in terms of basic soil research. Previously no work was reported either on the population dynamics of the soil bacteria or on the relationship between soil microflora and soil physicochemical properties of coastal areas of Digha. In this context, the present study has been carried out to determine the diversity of soil bacteria and the physicochemical properties of the soil of different villages of the coastal areas of Digha, West Bengal, India.

## Materials and methods

### Soil collection

The soil samples were collected from nine village areas of Midnapore coastal belt, West Bengal, India: [Padima (21°37′39.99″N, 87°29′28.81″E), Jatimati (21°37′40.90″N, 87°29′46.66″E), Chanpabani (21°37′39.89″N, 87°30′22.64″E), Palsandapur (21°37′30.36″N, 87°30′04.46″E), Bhagibaharampur (21°37′36.48″N, 87°30′44.42″E), Duttapur (21°36′57.78″N, 87°29′29.71″E), Gadadharpur (21°37′05.81″N, 87°30′04.41″E), Gobindabasan (21°37′36.23″N, 87°31′18.36″E) and Somaibasan (21°37′50.71″N, 87°32′11.37″E)] during November 2013–February 2014. The topmost soil (1 cm) was scrapped off and then about 100 g of soil from each area was collected in sterile polythene bags sealed with rubber bands.

### Soil analysis

The soil samples were taken to the Microbiology and Parasitology Research Laboratory, The University of Burdwan, for both physico-chemical and microbial analysis.

To determine the heterotrophic viable aerobic bacterial population, soil samples were diluted up to 10^−5^ and a 10 μl soil suspension (10^−5^) was mixed with 100 ml nutrient agar (peptone 5 g/l, beef extract 3 g/l, agar 2 g/l, pH 7) and incubated at 30 ± 1 °C in the BOD incubator. To determine different groups of bacteria separately, 10 μl of soil suspension (10^−4^) was mixed with 100 ml of different specific media distributed in five plates and incubated at 30 ± 1 °C in the BOD incubator. Soil suspension was pasteurized at 60 °C for 30 min for enrichment culture of the spore formers. The starch-hydrolyzing bacteria were enumerated by incubating the soil on starch agar media for 24 h and counting the number of bacterial colony producing halo zone after flooding with Gram’s iodine. The nitrifying bacteria were recorded after 5–30 days (5 day intervals) from the date of incubation. But all other groups of bacterial populations were counted after 3-day incubation. The aerobic heterotrophic and spore-forming bacteria were enumerated using nutrient agar media following the standard methods. To visualize Gram-negative bacteria, crystal violet (peptone 5 g/l, beef extract 3 g/l, lactose 10 g/l, crystal violet 0.0033 g/l, agar 15 g/l, pH 6.8 ± 0.1) was added to the medium before plating. *Pseudomonas* population were enumerated by incubating the soil suspension on *Pseudomonas* isolation agar [peptic digest of animal tissue 20 g/l, magnesium chloride 1.4 g/l, potassium sulfate 10 g/l, triclosan (Irgasan) 0.025 g/l, Agar 13.6 g/l, pH 7.0] for 72 h. Nitrifying bacteria was enumerated on Winogradsky’s medium containing (NH_4_)_2_SO_4_ (1.0 g/l) and the colonies were visualized (pink colour) by flooding the plates with sulphanilic acid reagent. The inorganic phosphate solubilizing bacteria were determined from the halo zone formation around the colonies on the insoluble phosphate [Ca_3_(PO_4_)_2_] containing medium. The asymbiotic nitrogen-fixing bacteria were counted on nitrogen-free medium (Pelczar et al. 1957; Lacey 1997; Chatterjee et al. 2014). The physico-chemical parameters of the soil samples were measured following the standard methods (Issac and Johnson 1984; Sumner and Miller 1996; Evangelou 1998).

### Statistical analyses

Shannon-Wiener Index and Simpson Index were calculated to determine the species diversity and richness of the bacterial population of the soil samples collected from coastal areas of Digha. The data obtained on the different groups of bacteria present in the soil samples of Digha were subjected to agglomerative hierarchial cluster analysis. Principal component analysis (PCA) was done to represent a relationship between the various groups of bacteria (Manly 1994; Zar 1999; Kinnear and Gray 2000).

## Result

**Fig. 1:**
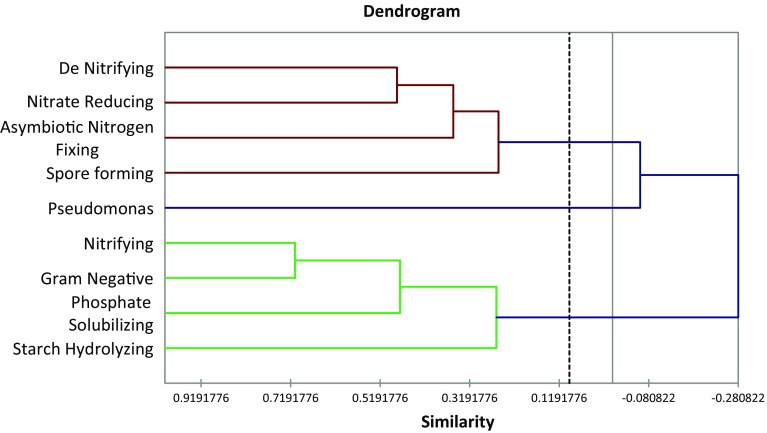
Agglometric hierarchical cluster analysis of the soil bacterial isolates of different villages of Digha, West Bengal

**Fig. 2:**
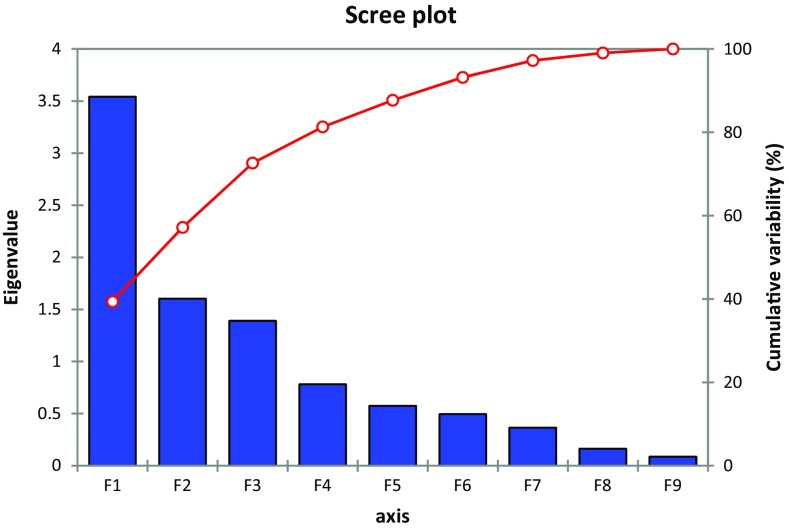
Scree plot derived from PCA regarding the bacterial isolates of the collected soil samples of coastal areas of Digha, West Bengal, India

**Fig. 3:**
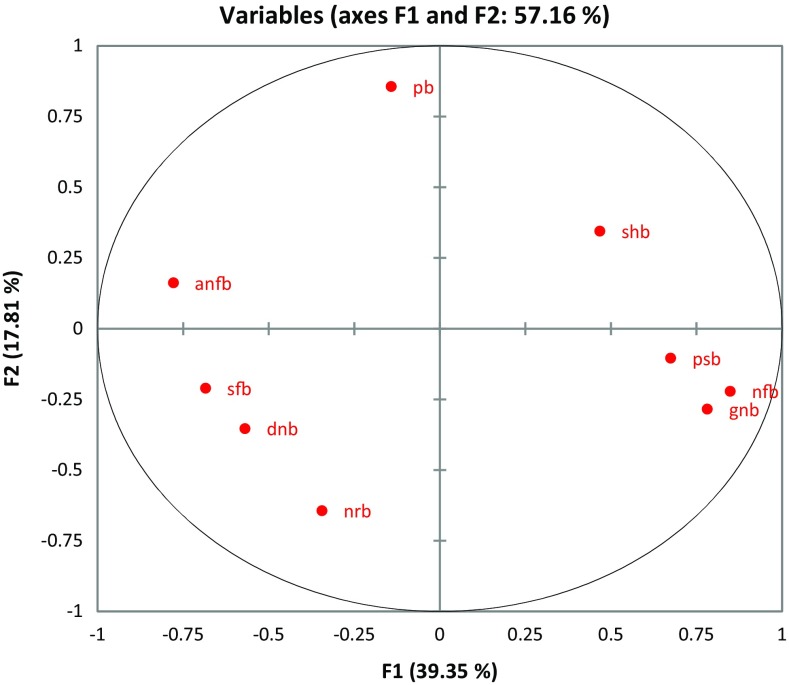
Quadrant distribution of the soil bacterial isolates of the coastal areas of Digha, West Bengal, via PCA. *anfb* asymbiotic nitrogen-fixing bacteria, *pb* *Pseudomonas* bacteria, *shb* starch-hydrolyzing bacteria, *psb* phosphate-solubilising bacteria, *nfb* nitrifying bacteria, *gnb* Gram-negative bacteria, *nrb* nitrate-reducing bacteria, *dnb* denitrifying bacteria, *sfb* spore-forming bacteria

In the village areas of the coastal areas of Digha, the aerobic heterotrophic, Gram negative, spore-forming, starch-hydrolyzing, *Pseudomonas*, nitrate-reducing, de nitrifying, asymbiotic N_2_ fixing, nitrifying, phosphate-solubilizing bacterial populations ranged from 1.22 to 2.67 × 10^6^, 0.09–1.63 × 10^5^, 1.53–3.68 × 10^5^, 2.22–4.06 × 10^5^, 0.02–0.04 × 10^5^, 0.35–1.33 × 10^5^, 0.07–0.82 × 10^5^, 0.58–2.50 × 10^5^, 0.13–2.35 × 10^5^, 0.05–1.9 × 10^5^ cfu/g dry soil, respectively (Table 1). In soil samples of Palsandapur, aerobic heterotrophic population, Gram-negative and phosphate-solubilising bacterial population were higher than in other regions of the study area. The spore-forming bacterial population was found to be higher in the soil samples of Chanpabani than that of Padima, Jatimati, Palsandapur, Bhagibaharampur, Duttapur, Gadadharpur, Gobindabasan and Somaibasan. The soil samples of Gadadharpur area contained higher number of starch-hydrolyzing, *Pseudomonas* and asymbiotic nitrogen-fixing bacteria than other villages of the present study area. Nitrate-reducing and de nitrifying bacterial population were comparatively higher in Jatimati village in respect of other villages of the study sites. In soil samples of Gobindabasan area, the nitrifying bacterial population was higher than that in Padima, Jatimati, Chanpabani, Palsandapur, Bhagibaharampur, Duttapur, Gadadharpur and Somaibasan area. The organic carbon content of the soil samples ranged from 0.61 to 0.93 % and found to be higher in soil samples of Palsandapur (0.93 %) than other villages of the study area. The available nitrogen in the soils of the study area varied from 11.2 to 29.5 mg/kg and was highest in Gadadharpur area (29.5 mg/kg). The available phosphate content in the soil samples of the coastal areas of Digha ranged from 230.8 to 503.09 mg/kg. In the Palsandapur area, the phosphate content was much higher (503.09 mg/kg) than other villages of our study area. The soil salinity ranged between 5.2 and 23.2 ds/m in the study area. The soil salinity was found to be lower in Palsandapur area and was comparatively higher in Gobindabasan area (Table 2). The results of the one-way ANOVA revealed significant variations (*p* < 0.05) in the microbial diversity with respect to different locations of the study site. Agglometric hierarchial cluster analysis (AHC) in respect of the number of different bacterial groups in different places of the study area showed that the denitrifying, nitrate-reducing, asymbiotic nitrogen-fixing and spore-forming bacteria formed a cluster in respect of their distribution pattern while *Pseudomonas* differed from them forming another cluster. Nitrifying, Gram-negative, phosphate-solubilising and starch-hydrolyzing bacteria formed a different cluster (Fig. 1). From principal component analysis (PCA) three components were extracted having the Eigen values of 3.541, 1.603 and 1.391, respectively, that explained more than 72 % (component 1—39.35 %, component 2—17.8 % and component 3—15.45 %) of the variance on the microbial abundance in the soil samples of the coastal areas of Digha (Table 3; Fig. 2). The bacterial groups were plotted on quadrant plot and among the four quadrants, starch-hydrolyzing bacteria were on the first quadrant, the second quadrant comprised of phosphate-solubilising, Gram-negative and nitrifying bacterial groups. Spore-forming, nitrate-reducing and denitrifying bacteria were in the third quadrant and the fourth quadrant contained *Pseudomonas* and asymbiotic nitrogen-fixing bacteria (Fig. 3). Shannon-Wiener and Simpson Index of the study areas ranged from 1.56 to 1.88 and 3.85–5.73, respectively. Jatimati showed comparatively higher diversity index among the villages of the study area (Table 4).

**Table 1:** Population dynamics [(cfu ± SE)/g dry soil] of different microbes in soil samples of coastal areas of Digha, West Bengal, India

Bacterial population	Padima	Jatimati	Chanpabani	Palsandapur	Bhagibaharampur	Duttapur	Gadadharpur	Gobindabasan	Somaibasan
Heterotrophic (10^6^)	1.53 ± 0.06	1.54 ± 0.024	1.36 ± 0.034	2.67 ± 0.037	1.58 ± 0.036	2.38 ± 0.032	1.51 ± 0.059	1.22 ± 0.062	2.52 ± 0.067
Gram-negative (10^5^)	1.27 ± 0.05	0.656 ± 0.034	0.094 ± 0.003	1.63 ± 0.037	0.456 ± 0.047	0.722 ± 0.055	0.544 ± 0.038	1.3 ± 0.069	1.62 ± 0.06
Spore-forming (10^5^)	3.29 ± 0.08	2.9 ± 0.1	3.68 ± 0.068	2.51 ± 0.166	3.18 ± 0.091	3.66 ± 0.067	3.04 ± 0.138	1.53 ± 0.112	3.53 ± 0.122
Starch-hydrolyzing (10^5^)	3.30 ± 0.08	3.39 ± 0.048	3.33 ± 0.05	3.89 ± 0.079	3.4 ± 0.097	2.22 ± 0.062	4.07 ± 0.098	3.83 ± 0.096	3.18 ± 0.148
Pseudomonas (10^5^)	0.020 ± 0.002	0.016 ± 0.003	0.043 ± 0.002	0.026 ± 0.003	0.024 ± 0.005	0.024 ± 0.003	0.046 ± 0.003	0.037 ± 0.004	0.021 ± 0.004
Nitrate-reducing (10^5^)	0.48 ± 0.008	1.33 ± 0.047	0.576 ± 0.016	0.402 ± 0.01	0.508 ± 0.013	0.508 ± 0.019	0.373 ± 0.012	0.351 ± 0.015	0.457 ± 0.011
De nitrifying (10^5^)	0.80 ± 0.05	0.822 ± 0.052	0.556 ± 0.06	0.072 ± 0.006	0.344 ± 0.038	0.323 ± 0.012	0.622 ± 0.068	0.264 ± 0.014	0.544 ± 0.056
Asymbiotic nitrogen-fixing (10^5^)	1.61 ± 0.05	2.02 ± 0.08	2.29 ± 0.123	0.6 ± 0.029	0.733 ± 0.062	2.31 ± 0.096	2.5 ± 0.133	1.02 ± 0.032	0.578 ± 0.028
Nitrifying (10^5^)	1.20 ± 0.06	1.49 ± 0.092	0.29 ± 0.011	2.11 ± 0.075	0.544 ± 0.029	0.133 ± 0.012	0.367 ± 0.029	2.36 ± 0.103	1.31 ± 0.087
Phosphate solubilizing (10^5^)	0.46 ± 0.024	0.299 ± 0.012	0.081 ± 0.007	1.9 ± 0.133	0.251 ± 0.01	0.311 ± 0.009	0.07 ± 0.007	0.24 ± 0.01	0.054 ± 0.006

Results are mean of nine replication ± SE

*cfu* colony-forming unit

**Table 2:** Physico-chemical parameters of the soil samples collected from different areas of coastal areas of Digha, West Bengal

Place	Organic carbon (%)	Nitrogen (mg/kg)	Phosphate (mg/kg)	Salinity (ds/m)
Padima	0.71	21.32	245.26	9.7
Jatimati	0.85	29.4	244.78	10.3
Chanpabani	0.68	20.12	230.91	11.2
Palsandapur	0.93	18.75	503.09	5.2
Bhagibaharampur	0.83	12.6	243.81	10.6
Duttapur	0.90	16.5	244.92	20.8
Gadadharpur	0.85	29.5	230.75	15.2
Gobindabasan	0.61	11.2	243.8	23.2
Somaibasan	0.89	23.2	230.8	18.7

**Table 3:** Eigenvalues extracted from the Principal Component Analysis

	F1	F2	F3
Eigenvalue	3.541	1.603	1.391
Variability (%)	39.349	17.808	15.456
Cumulative %	39.349	57.156	72.613

**Table 4:** Soil bacterial diversity indices of different villages of coastal areas of Digha, West Bengal, India

Place	Shannon-Wiener Index	Simpson’s index
Padima	1.8614581	5.4290517
Jatimati	1.8814364	5.726694
Chanpabani	1.5576826	3.9089861
Palsandapur	1.7972952	5.2850797
Bhagibaharampur	1.6336093	3.8501342
Duttapur	1.6592783	4.231307
Gadadharpur	1.6203932	4.0984608
Gobindabasan	1.7516001	4.6757094
Somaibasan	1.7141768	4.5963072

## Discussion

Physicochemical properties of soil, such as textures, water holding capacity, pH, organic matter content, etc. can influence microbial community structure by providing specific habitats for specific microorganisms (Chatterjee et al. 2014). The soil-inhabiting microorganisms play a vital role in regulating soil function by contributing to soil structure formation (Wright and Upadhyaya 1998; Dodd et al. 2000), plant nutrition (George et al. 1995; Timonen et al. 1996), plant health (Srivastava et al.^1996^; Filion et al. 1999) and soil fertility (Yao et al. 2000; O’Donnell et al. 2001). So quantitative and qualitative assay of soil microbes is an essential part in characterization of soil (Arunachalam and Arunachalam 2000). Ranjard and Richaume (2001) have done such characterization in France. Das et al. (1997) and Das and Dangar (2008) have studied the microbial diversity in the soils of Himalayan region and saline soils of rice field of coastal Orissa. In the present study, viable plate count method was adopted to assess the microbial diversity in the soil samples. In coastal areas of Digha, the aerobic heterotrophic bacterial population was quite lower than that in forest soil, agricultural and botanic soils of different parts of India. It was previously recorded that the heterotrophic bacterial population was found to be lower in sandy soils due to lower organic content than clay or humus soils (Kaur and singh 2000). The higher salinity might also be the reason of lower density of heterotrophic bacteria in coastal saline soils. Gram-positive bacterial Population was generally higher than Gram-negative bacterial Population, probably due to the ability of Gram-positive bacteria to form endospores and develop other stress response mechanisms (Hecker et al. 2007). Aerobic endospore-formers were found to be important in the soil nutrient cycle, such as the nitrogen cycle was influenced by denitrifiers, nitrogen fixers and organic nitrogen degraders; so as the sulphur cycle by sulphur oxidizers; and in transformation of other soil nutrients, such as manganese reduction. The abilities of the spore-forming bacteria to break down cellulose, hemicelluloses and pectins would suggest their roles in mineralization of plant material and humic material, while chitinase activity might help in degradation of fungal cell walls and insect exoskeletons (Mandic-Mulec and James 2011). In this study it was observed that spore-forming bacteria clustered with denitrifying, nitrate reducing with asymbiotic nitrogen-fixing bacteria (Fig. 1). May be the spore-forming isolates provided nutrients that help these other organisms to grow. According to van Gestel et al. (1996), the vicinity between microbes, organic matter and clay is required for the survival of microbes, in which the organic matter and clay particles provide substrates to live in and nutrients to grow and function. In a study in Orissa, Dangar et al. (2010) found that denitrifying bacterial population was high in flooded or water saturated soil. Probably moisture content was inversely and organic content was directly related to the density of microbial population (Pankhurst et al. 1996; Reichardt et al. 2001). In Palsandapur area, the phosphate content was much higher than in other places of the study sites (Table 2) that might favour the growth of phosphate-solubilising bacteria in this soil. The higher nitrogen content in Gadadharpur soil sample might be due to the high density of asymbiotic nitrogen-fixing bacteria in that soil. Asymbiotic nitrogen-fixing bacteria could fix the nitrogen as nitrate which increased the nitrogen availability in soil. Shannon-Wiener Index and Simpson Index showed that Jatimati area was most diversified area in respect of soil microbial diversity among all the studied areas. The higher organic carbon, nitrogen and phosphate content of this area provided an apt environment for the bacterial communities. The ANOVA result indicated a variation in the relative density of the microbes present in the soils sampled during the study (*p* < 0.05). This variation was dependent on the microhabitat they were provided in different locations of the study area. The AHC analysis revealed the correlation between the bacterial groups in terms to their relative density which was further justified by PCA. The PCA helped to understand the correlation between the different bacterial groups based on their abundance in different soil samples of the coastal areas of Digha. Gomoryova et al. (1999) assessed the distribution of functional groups of microbes via BIOLOG analysis, and his further analysis concluded that two environmental variables, i.e. tree influence potential and organic carbon content of soil, significantly influenced the microbial composition of a particular soil.

## Conclusion

This study can be considered as a promising start for identification and distribution of various bacterial families in different soil samples of the coastal areas of Digha, West Bengal, India. Though this is a preliminary work regarding the assessment of bacterial diversity, it can be helpful for further specific identification and characterization of the bacterial communities inhabiting the coastal area. The diversity of spore-forming bacteria in this area increases the chance of the availability of potential insect pathogens which may be helpful in the studies regarding biocontrol.
